# Newtonian versus Special-Relativistic Statistical Predictions for Low-Speed Scattering

**DOI:** 10.1371/journal.pone.0048447

**Published:** 2012-11-12

**Authors:** Shiuan-Ni Liang, Florentino Borondo, Boon Leong Lan

**Affiliations:** 1 School of Science, Monash University, Selangor, Malaysia; 2 Departamento de Química and Instituto Mixto de Ciencias Matemáticas CSIC-UAM-UC3M-UCM, Universidad Autónoma de Madrid, Cantoblanco, Madrid, Spain; University of Nottingham, United Kingdom

## Abstract

The statistical predictions of Newtonian and special-relativistic mechanics, which are calculated from an initially Gaussian ensemble of trajectories, are compared for a low-speed scattering system. The comparisons are focused on the mean dwell time, transmission and reflection coefficients, and the position and momentum means and standard deviations. We find that the statistical predictions of the two theories do not always agree as conventionally expected. The predictions are close if the scattering is non-chaotic but they are radically different if the scattering is chaotic and the initial ensemble is well localized in phase space. Our result indicates that for low-speed chaotic scattering, special-relativistic mechanics must be used, instead of the standard practice of using Newtonian mechanics, to obtain empirically-correct statistical predictions from an initially well-localized Gaussian ensemble.

## Introduction

The standard practice in dynamics is to use Newtonian mechanics to study the motion of low-speed (i.e., much smaller than the speed of light) particles, instead of using the special-relativistic theory. This practice is rooted in the conventional belief [1]–[3] that the dynamics predicted by special-relativistic mechanics for a low-speed system is always well-approximated by the dynamics predicted by Newtonian mechanics from the same parameters and initial conditions. Special-relativistic dynamics of nonlinear systems have been studied in the past – examples include the relativistic kicked harmonic oscillator [4]–[7], the relativistic kicked rotor [8] and the relativistic hydrogen-like atom [9]. However, the conventional belief about the relationship between Newtonian and special-relativistic dynamics at low speed has not been critically scrutinized until recently.

In a numerical study of a low-speed model Hamiltonian system [10], [11], one of us found that the Newtonian trajectory does not always remain close to the special-relativistic trajectory as expected – the two trajectories eventually become completely different regardless of whether the trajectories are chaotic or non-chaotic. The breakdown of agreement between the Newtonian and special-relativistic trajectories is, however, much faster in the chaotic case compared to the non-chaotic, since the difference between the two trajectories grows exponentially in the former case but linearly in the latter case. Similar rapid breakdown of agreement was found numerically in other low-speed systems, in particular, a model dissipative system [12] and a model scattering system [13]. For the scattering system in [13], the rapid breakdown of agreement was found to be due to a sufficiently-long exponential growth of the difference between the two trajectories in the scattering region when the scattering is chaotic.

In this paper, we extend the comparison of the Newtonian and special-relativistic single-trajectory predictions for the low-speed model scattering system presented in Ref. [13] to a comparison of statistical quantities which are calculated from the same parameters and initial ensemble of trajectories. The statistical quantities we will focus on are the mean dwell time, transmission and reflection coefficients, and the position and momentum means and standard deviations. The dwell time is, for each trajectory in the ensemble, defined as (*t*_out_ – *t*_in_) where *t*_in_ is the time when the particle first enters the scattering region and *t*_out_ is the time when the particle subsequently first exits the scattering region. The transmission coefficient (reflection coefficient) is defined as the ratio of the number of transmitted (reflected) particles to the total number of particles in the ensemble. A comparison of the Newtonian and special-relativistic statistical predictions for a low-speed scattering system has not yet been done. In the recent numerical study [14] by two of us where the statistical predictions of the two theories were compared for the low-speed model Hamiltonian system studied in [10], it was not possible to compare the mean dwell time and also the transmission and reflection coefficients because the system is spatially bounded, not a scattering system which is spatially unbounded.

**Figure 1:**
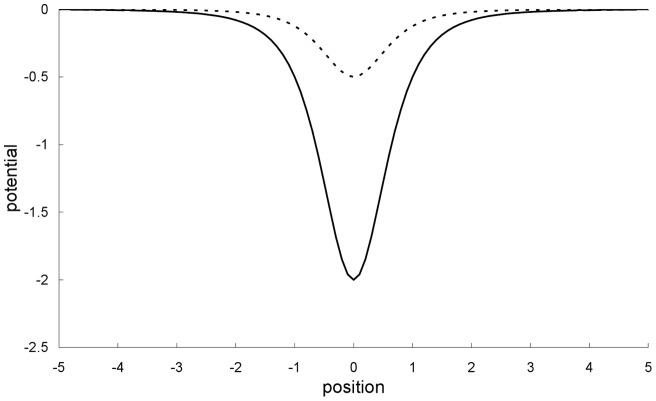
Scattering potential well. Scattering potential well for *V*_0_ = 8 and *β* = 4 (solid line), and *V*_0_ = 2 and *β* = 4 (dotted line).

The model scattering system we have chosen to study allows sufficiently-accurate calculation of the statistical quantities because the time-evolution of each trajectory in the ensemble is described by an exact analytical map in both the Newtonian and special-relativistic frameworks. Details of the model scattering system and calculations are given next, followed by the presentation and discussion of the results, and, finally, our concluding remarks.

## Methods

The scattering system consists of a particle of rest mass *m*_0_ moving in the one-dimensional potential well introduced by Beeker and Eckelt [15]:

**Figure 2:**
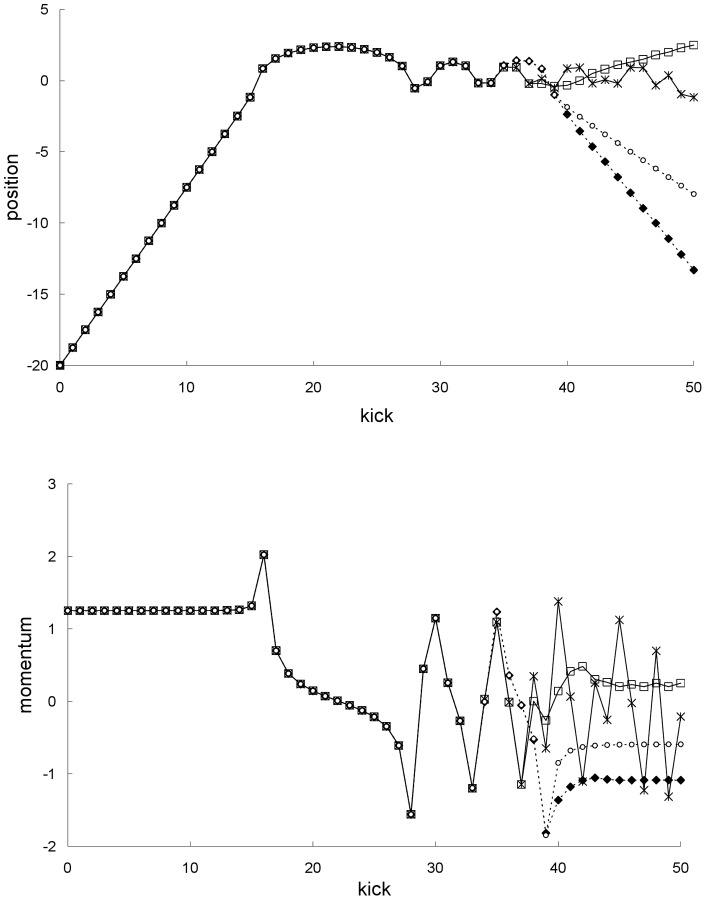
Single and mean trajectories for the first example. Newtonian single trajectory (asterisks), Newtonian mean trajectory (squares), special-relativistic single trajectory (circles) and special-relativistic mean trajectory (diamonds) for the chaotic scattering case in the first example discussed in the text: positions (top plot) and momentums (bottom plot).



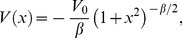
(1)which is periodically turned on only for an instant of time. The potential well is characterized by two parameters *V*_0_ and *β*, where *V*_0_/*β* determines the depth of the well and *β* determines its asymptotic behavior.

**Figure 3:**
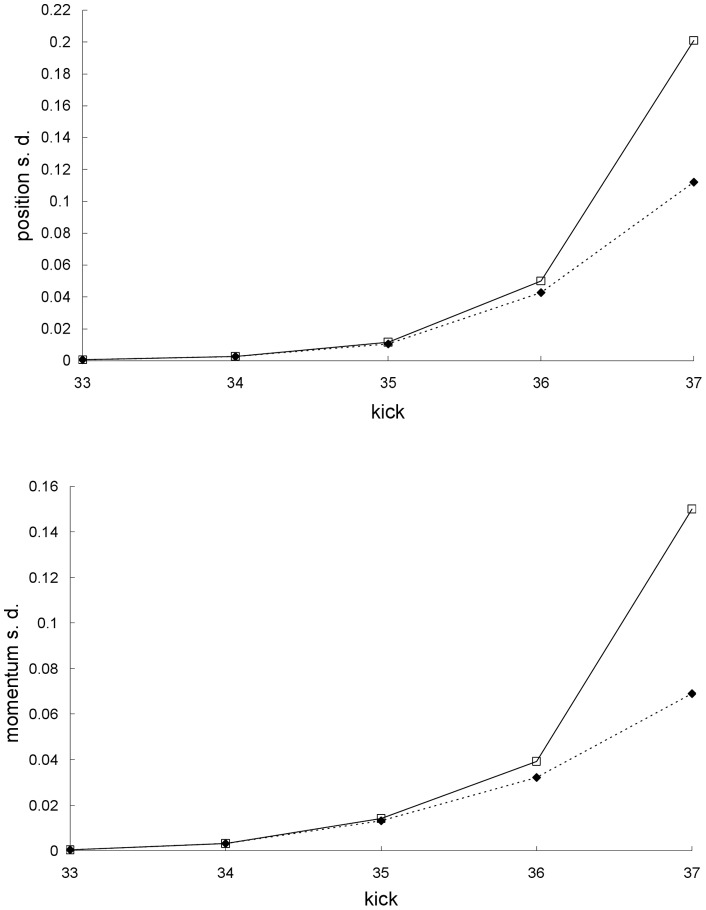
Standard deviations for the first example. Newtonian (squares) and special-relativistic (diamonds) position standard deviations (top plot) and momentum standard deviations (bottom plot) for the chaotic scattering case in the first example. The Newtonian and special-relativistic standard deviations are not plotted before kick 33 because they are close to each other.

**Figure 4:**
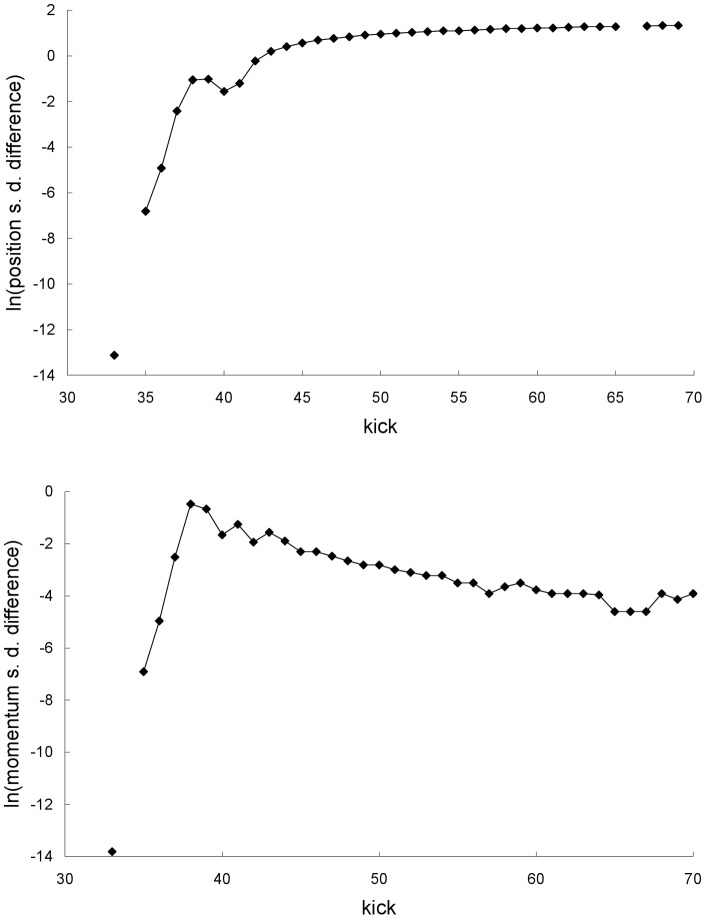
Difference between the standard deviations for the first example. Difference between the Newtonian and special-relativistic standard deviations – for position (top plot), and momentum (bottom plot) – for the chaotic scattering case in the first example. The standard-deviation differences before kick 33 and at kick 34 are not shown because they cannot be resolved with the accuracy of our calculations. After the exponential growth, which ends at kick 38, the position-standard-deviation difference grows linearly from kick 50 onwards and the momentum-standard-deviation difference is essentially constant from kick 60 onwards.

The Newtonian equations of motion for this periodically-delta-kicked scattering system are easily integrated exactly [15] to yield a mapping for the position *x* and momentum *p* from just before the *n*th kick to just before the (*n*+1)th kick:

**Figure 5:**
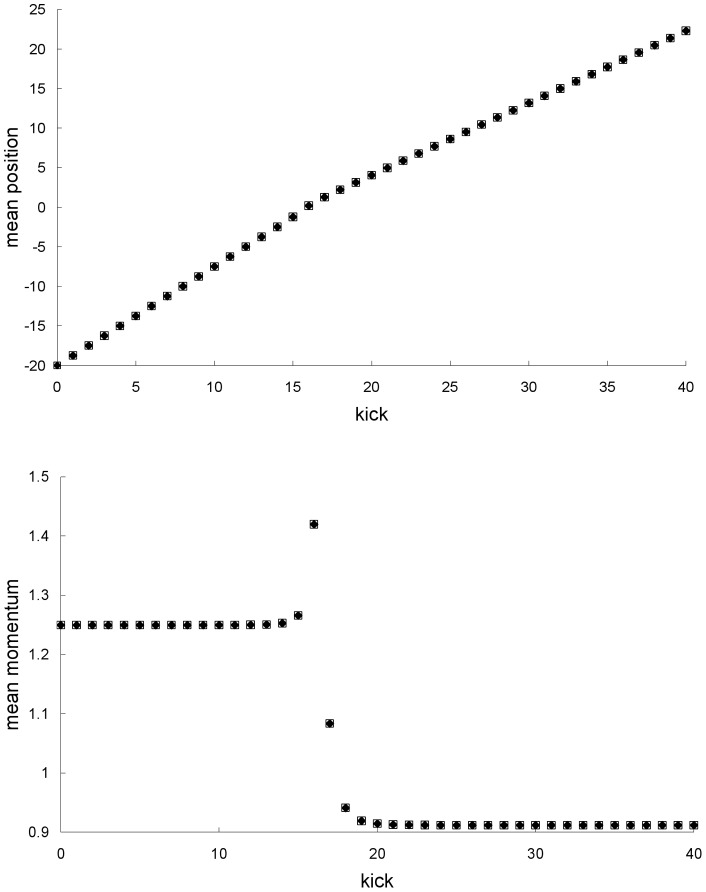
Mean trajectories for the third example. Newtonian (squares) and special-relativistic (diamonds) mean positions (top plot) and mean momentums (bottom plot) for the non-chaotic scattering case in the third example discussed in the text.



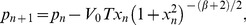
(2)

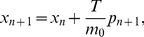
(3)


where *T* is the kicking period.

**Figure 6:**
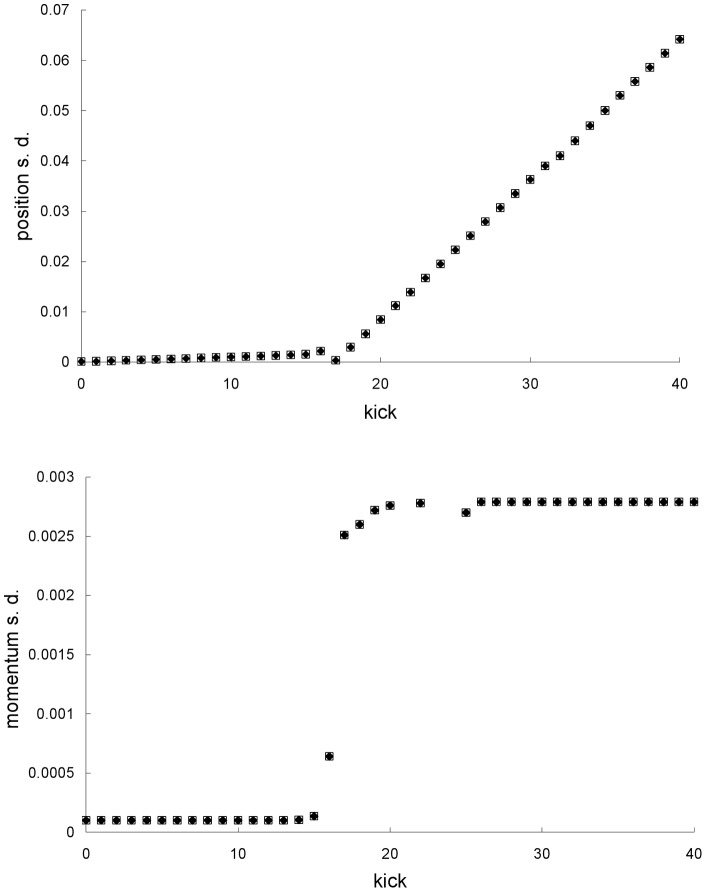
Standard deviations for the third example. Newtonian (squares) and special-relativistic (diamonds) position standard deviations (top plot) and momentum standard deviations (bottom plot) for the non-chaotic scattering case in the third example.

The corresponding special-relativistic equations of motion are also easily integrated exactly [13] to produce a mapping for the position *x* and momentum *p* from just before the *n*th kick to just before the (*n*+1)th kick:
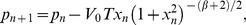
(4)

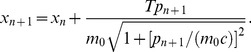
(5)


For both theories, we consider an initially Gaussian ensemble of trajectories centered at the mean values <*x*> and <*p*>, and with standard deviations *σ_x_* and *σ_p_*:
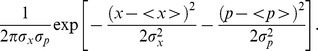



Each trajectory in the Newtonian (special-relativistic) ensemble is time-evolved using the map given by Eqs. (2) and (3) [Eqs. (4) and (5)]. For both theories, each statistical quantity is calculated by averaging over the ensemble of trajectories. We ensured that the statistical quantity from each theory is numerically accurate in the following way. First, the statistical quantity is calculated using 10^6^ trajectories, where its accuracy is determined by comparing the less accurate 30-significant-figure calculation with the more accurate quadruple-precision (35 significant figures) calculation. For example, at a particular kick, if the statistical quantity from the 30-significant-figure calculation is 5.1234567…, and the same statistical quantity from the quadruple-precision calculation is 5.1234568…, then the accurate value for that statistical quantity, based on 10^6^ trajectories, is 5.123456. The statistical quantity is then recalculated using 10^7^ trajectories where its accuracy is determined in the same manner. Finally, the accuracy of the statistical quantity is determined by comparing the less accurate 10^6^-trajectories calculation with the more accurate 10^7^-trajectories calculation. We used *m*_0_ = 1, *T* = 1, and *c* = 10^5^ in all of our calculations.

## Results

In this section we present and discuss four examples to illustrate the general results. In all cases, the mean speed is low, only 0.001% of the speed of light.

In the first example, the parameters of the scattering potential well are *V*_0_ = 8 and *β* = 4. The corresponding potential profile is plotted in Figure 1. For these parameters, the scattering is chaotic, i.e., the scattering function has intertwining regular and irregular intervals down to all scales, from both the Newtonian [15] and special-relativistic [13] perspectives. The means and standard deviations of the initially Gaussian ensemble are <*x*> = −20, <*p*> = 1.2497 and *σ_x_* = *σ_p_* = 10^−11^. This initially localized ensemble is far from and to the left of the scattering region ranging from *x* = −4 to *x* = 4.

Figure 2 shows that the Newtonian mean trajectory, i.e., mean position and mean momentum, agrees with the special-relativistic mean trajectory for the first 35 kicks. The two mean trajectories are completely different, however, from kick 36 onwards. This breakdown of agreement can be understood as follows. The Newtonian (special-relativistic) mean trajectory is, see Figure 2, well-approximated by the Newtonian (special-relativistic) single trajectory with the same initial conditions until the Newtonian (special-relativistic) ensemble is delocalized in phase space at kick 38 (kick 39). In other words, at kick 36, the Newtonian and special-relativistic mean trajectories are still well-approximated by the corresponding single trajectories. Since the agreement between the Newtonian and special-relativistic single trajectories breaks down at kick 36 (see Figure 2), the agreement between the Newtonian and special-relativistic mean trajectories therefore also breaks down at the same kick. Furthermore, the breakdown of agreement between the Newtonian and special-relativistic mean trajectories is rapid because the difference between them grows exponentially in the scattering region, like the growth of the difference between the Newtonian and special-relativistic single trajectories shown previously in Ref. [13].

Figure 3 shows that the agreement between the Newtonian and special-relativistic standard deviations also breaks down at kick 36. Figure 4 shows that this rapid breakdown of agreement is due to the exponential growth of the difference between the Newtonian and special-relativistic standard deviations up to kick 38 while the ensembles are still in the scattering region.

The mean dwell time, transmission coefficient and reflection coefficient predicted by the two theories are also, remarkably, very different. Indeed, the Newtonian mean dwell time is 32.9 kicks, while the corresponding special-relativistic value is only 30.3 kicks. Even more striking is the difference in the transmission coefficients, since the Newtonian value of 0.57 is more than two times the special-relativistic value of 0.24. Similarly, for the reflection coefficient, the special-relativistic value of 0.75 is about two times the Newtonian value of 0.42.

In the second example, the scattering is also chaotic. All the parameters are the same as in the first example except that a broader initial Gaussian ensemble, both in position and momentum with *σ_x_* = *σ_p_* = 10^−7^, is used. In contrast to what happened in the previous example, there is no breakdown of agreement between the position and momentum means and standard deviations predicted by the two theories. In this example, when the Newtonian and special-relativistic ensembles delocalized in phase space at kick 32, the Newtonian and special-relativistic mean trajectories are still close to one another because the agreement between the Newtonian and special-relativistic single trajectories with the same initial conditions only breaks down sometime later at kick 36. The Newtonian and special-relativistic standard deviations at kick 32 are also still close to one another. Hence, since the Newtonian and special-relativistic delocalized phase-space distributions are close to one another at kick 32, the subsequent predictions of the means and standard deviations by the two theories continue to be close. Furthermore, the other statistical quantities predicted by the two theories are also close, in particular, they agree to at least 2 significant figures: the two theories predict 25 kicks for the mean dwell time, 0.39 for the transmission coefficient and 0.60 for the reflection coefficient.

These two examples of chaotic scattering illustrate that the statistical predictions of the two theories are radically different if the initially Gaussian ensemble is well, i.e., sufficiently, localized in phase space such that the Newtonian and special-relativistic ensembles delocalize *after* the agreement between the Newtonian and special-relativistic single trajectories, with the same initial conditions as the Newtonian and special-relativistic mean trajectories, breaks down.

In the third example, the parameters of the scattering potential well are *V*_0_ = 2 and *β* = 4 – the corresponding potential profile has also been plotted in Figure 1. For these values of the parameters, the scattering is non-chaotic, i.e., the scattering function varies regularly from both the Newtonian [15] and special-relativistic [13] perspectives. The means and standard deviations of the initially Gaussian ensemble are <*x*> = −20, <*p*> = 1.2497 and *σ_x_* = *σ_p_* = 10^−4^. This choice initially localize the ensemble far from and to the left of the scattering region, which is in the range *x* = −3.5 to *x* = 3.5. In this case, the transmission and reflection coefficients predicted by the two theories are the same, and equal to 1 and 0, respectively. Both the Newtonian and special-relativistic ensembles are still localized in phase space when they are far away from the scattering region on the other side at *x
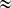
*20 at kick 40. Figure 5 shows that when the Newtonian and special-relativistic ensembles are far away from the scattering region on the other side, the Newtonian and special-relativistic mean trajectories are still close to one another – this is because they are well-approximated by the corresponding single trajectories (with the same initial conditions) which are still also close to one another. The Newtonian and special-relativistic standard deviations are also close to one another – see Figure 6. The mean dwell times from the two theories are also the same: 6 kicks.

In the fourth and final example, the scattering is also non-chaotic. All the parameters are the same as in the third example except that the initial Gaussian ensemble is broader in both position and momentum with *σ_x_* = *σ_p_* = 10^−2^. In contrast to the previous example, the Newtonian and special-relativistic ensembles are already delocalized in phase space at kick 17 in the scattering region. The two ensembles are still close to one another when they delocalize, which means that there is no subsequent breakdown of agreement between the means and the standard deviations predicted by the two theories for the position and momentum. Furthermore, the transmission and reflection coefficients predicted by the two theories are the same, 1 and 0, respectively. The two predictions for the mean dwell time agree to at least 3 significant figures, in particular, 5.64 kicks.

The third and fourth examples above illustrate that if the scattering is non-chaotic, there is no breakdown of agreement between the statistical predictions of the two theories.

## Discussion

We have shown that the Newtonian and special-relativistic statistical predictions for the mean dwell time, transmission and reflection coefficients, and the position and momentum means and standard deviations, which are calculated from an initially Gaussian ensemble of trajectories, for a low-speed scattering system are radically different if the scattering is chaotic and the initial ensemble is well localized in phase space. In contrast, there is no breakdown of agreement between the two statistical predictions if the scattering is non-chaotic.

Our finding raises an important fundamental question in physics: When the Newtonian and special-relativistic statistical predictions – particularly the mean dwell time, and transmission and reflection coefficients – are completely different for a low-speed scattering system, which of the two predictions is empirically correct? Since special relativity continues to be successfully tested [16]–[18] in recent times, we expect the special-relativistic predictions to be the correct. This implies that Newtonian mechanics, which is (for example, see Ref. [19]) the standard theory used to study the dynamics of low-speed scattering systems, does not always yield statistical predictions that are empirically correct. Special-relativistic mechanics must be used instead to obtain empirically-correct statistical predictions when the low-speed scattering is chaotic and the initial Gaussian ensemble is well localized.
